# Determinants of Disease Presentation and Outcome during Cryptococcosis: The CryptoA/D Study 

**DOI:** 10.1371/journal.pmed.0040021

**Published:** 2007-02-06

**Authors:** Françoise Dromer, Simone Mathoulin-Pélissier, Odile Launay, Olivier Lortholary, the French Cryptococcosis Study Group

**Affiliations:** 1 Unité de Mycologie Moléculaire, Centre National de Référence Mycologie et Antifongiques, CNRS URA3042, Institut Pasteur, Paris, France; 2 Institut Bergonié, Centre Régional de Lutte Contre le Cancer du Sud-Ouest, Bordeaux, France; 3 Université Paris V, Hôpital Cochin, Service de Médecine Interne, CIC Vaccinologie Cochin–Pasteur, Paris, France; 4 Université Paris V–Hôpital Necker-Enfants Malades, Service des Maladies Infectieuses et Tropicales, Centre d'Infectiologie Necker-Pasteur, Paris, France; Duke University Medical Center, United States of America

## Abstract

**Background:**

Cryptococcosis is a life-threatening opportunistic fungal infection in both HIV-positive and -negative patients. Information on clinical presentation and therapeutic guidelines, derived mostly from clinical trials performed before introduction of highly active antiretroviral therapy in patients with cryptococcal meningoencephalitis, is missing data on extrameningeal involvement and infections by serotype D as opposed to serotype A of Cryptococcus neoformans.

**Methods and Findings:**

The prospective multicenter study CryptoA/D was designed in France (1997–2001) to analyse the factors influencing clinical presentation and outcome without the bias of inclusion into therapeutic trials. Of the 230 patients enrolled, 177 (77%) were HIV-positive, 50 (22%) were female, and 161 (72.5%) were infected with serotype A. Based on culture results at baseline, cryptococcosis was more severe in men, in HIV-positive patients, and in patients infected with serotype A. Factors independently associated with mycological failure at week 2 independent of HIV status were initial dissemination (OR, 2.4 [95% confidence interval (CI), 1.2–4.9]), high (>1:512) serum antigen titre (OR, 2.6 [1.3–5.4]), and lack of flucytosine during induction therapy (OR, 3.8 [1.9–7.8]). The three-month survival was shorter in patients with abnormal neurology or brain imaging at baseline, and in those with haematological malignancy.

**Conclusions:**

Thus sex, HIV status, and infecting serotype are major determinants of presentation and outcome during cryptococcosis. We propose a modification of current guidelines for the initial management of cryptococcosis based on systematic fungal burden evaluation.

## Abstract 2

Françoise Dromer and colleagues report that sex, HIV status, and infecting serotype are major determinants of cryptococcosis presentation and outcome.

## Editors' Summary

**Background.:**

For people with a healthy immune system, athletes' foot may be the only fungal infection they ever have. But individuals whose immune system has been damaged by infection with HIV or who are immune-suppressed after organ transplantation or cancer chemotherapy can develop cryptococcosis. This is a life-threatening infection caused by *Cryptococcus neoformans,* a fungus found in bird droppings that enters the human body through the lungs. The initial infection can go unnoticed, although a pneumonia-like disease sometimes develops. However, if the fungus spreads (disseminates) around the body it can cause other symptoms. The commonest of these is cryptococcal meningitis, a swelling of the membrane around the brain that can cause a stiff neck, headaches, and neurological symptoms such as palsies. For anyone with cryptococcal meningitis or severe pneumonia the current treatment guidelines recommend a two-week induction therapy with the antifungal drugs amphotericin B and flucytosine, followed by fluconazole for ten weeks.

**Why Was This Study Done?:**

Even when these guidelines are followed, cryptococcosis kills some people. The guidelines are based on data from clinical trials of antifungal drugs in HIV-positive patients done before there were effective treatments for HIV infections. Patients without meningitis and those with very severe disease were excluded from these trials. Also, it is known that two variants of C. neoformans cause cryptococcosis: variety *grubii* (also known as serotype A) and variety *neoformans* (serotype D). There is very little information about the impact of patient-related factors (such as sex, age, and HIV status) or the variety of C. neoformans on clinical presentation (the symptoms patients have when they first visit a doctor), therapeutic management, or disease outcomes in the real world; this information is needed to improve the treatment guidelines. In this nationwide study across France, the researchers have asked what factors influence clinical presentation and outcomes in HIV-positive and HIV-negative patients with cryptococcosis, and whether infections with the two varieties of C. neoformans behave similarly.

**What Did the Researchers Do and Find?:**

Patients were enrolled in the study when their first episode of cryptococcosis was confirmed by growing C. neoformans from blood, urine, or cerebrospinal fluid. Information was collected about their initial symptoms, which body sites were infected with fungus (their mycological status), and their treatment. Clinical and mycological data were also collected at two weeks and three months, and the blood levels of cryptococcal secreted molecules were measured to provide a “serum antigen titer,” a measure of fungal load. The researchers found that cryptococcosis was more severe at presentation in men, in HIV-positive patients, and in patients infected with C. neoformans variety *grubii*. Patients whose treatment failed at two weeks (judged by the continued presence of C. neoformans in culture of their body fluids) tended to have disseminated disease at presentation, to have high serum antigen titers, and not to have been given flucytosine during the initial round of therapy. Finally, three-month survival was worse in patients with abnormal neurology or brain imaging at the start of the study, or those with hematological malignancies (cancers that affect immune system cells).

**What Do These Findings Mean?:**

These findings provide new information on what determines the clinical presentation and outcome in patients with cryptococcosis. Because the study was done in France, these results will only apply to other countries where C. neoformans causes cryptococcosis—in some countries C. gattii can cause the disease. In addition, not all of the cryptococcosis cases that occurred in France during the study period were enrolled in the study, so it is possible that the infection might have had different characteristics in the missing patients. Nevertheless, the results of this study suggest that more patients would benefit from a two-week induction therapy with amphotericin B and flucytosine than currently recommended. This treatment, write the researchers, should be given to all patients with a high serum antigen titer, fungus in the blood, or initial infection at two separate body sites (in addition to those with cryptococcal meningitis or severe pneumonia for whom it is currently recommended). They also suggest that the fungal burden should be routinely determined in all patients so that their treatment can be adjusted if necessary.

**Additional Information.:**

Please access these Web sites via the online version of this summary at http://dx.doi.org/10.1371/journal.pmed.0040021

- A related *PLoS Medicine* Perspective article discusses current management of cryptococcosis
- MedlinePlus has encyclopedia pages on cryptococcosis
- US Centers for Disease Control and Prevention provides information on cryptococcosis
- Aidsmap has information on cryptococcosis and research into the disease provided by the charity NAM
- A Clinical Infectious Diseases article published in 2000 states the current treatment guidelines for the management of cryptococcosis

## Introduction

Cryptococcosis is a deadly opportunistic infection caused by an encapsulated yeast, Cryptococcocus neoformans. The major predisposing factor is the profound cellular immune defect caused by HIV infection, but other T cell–related immune defects and immunosuppressive treatments can also be associated [1]. Despite major advances in the treatment of HIV infection with highly active antiretroviral therapy (HAART), cryptococcosis is still diagnosed in Western countries [2–4]. In the southern part of Africa and in Southeast Asia, cryptococcosis remains a major concern, with up to 30% of AIDS patients presenting with C. neoformans infections [5,6].

C. neoformans variety *grubii* (serotype A) [7] has a worldwide distribution and is often described as the unique serotype causing infection in HIV-positive patients [8,9]. In Europe, however, variety *neoformans* (serotype D) is also responsible for infections in almost 20% of HIV-positive patients [10,11]. Variety *gattii* (serotypes B and C), recently raised to species level as Cryptococcus gattii [12], is usually limited to tropical and subtropical areas [13,14]. Several studies have shown that serotypes A and B differ in terms of host infected, geographic distribution, presentation of disease, outcome, and therapeutic management [15–17]. Variety *grubii* and variety *neoformans* were found to differ in terms of host infected and disease pattern in a retrospective analysis [10], although this finding will need confirmation in a prospective study.

Several prospective randomized therapeutic trials performed before the HAART era have been used to develop therapeutic guidelines for the management of HIV-positive patients with cryptococcosis [18]. With the contribution of earlier trials, guidelines were subsequently extrapolated for HIV-negative patients [19]. Description of disease presentation and outcome as well as factors influencing sterilisation of cerebrospinal fluid (CSF) or therapeutic failure have been published in HIV-negative [19–21] and -positive populations [22–24], mostly after analysis of therapeutic trials conducted in countries where variety *neoformans* (serotype D) is rarely recovered. Moreover, exclusion criteria for therapeutic trials usually reject the most severely affected patients and those without meningitis. Finally, with the exception of one recent study, which showed that male sex is an independent factor for mortality in HIV-negative patients with cryptococcal meningoencephalitis [21], no study so far has looked for the potential impact of host factors such as sex or HIV status, or of infecting variety *grubii* versus *neoformans* on clinical presentation, therapeutic management, and outcome of infection.

A nationwide multicenter prospective study (the CryptoA/D study) was thus designed in France in 1997—i.e. in the HAART era—to address two questions: First, what are the factors influencing clinical presentation and outcome in HIV-positive and -negative patients with cryptococcosis? And second, do infections by C. neoformans variety *grubii* and *neoformans* differ in these respects?

## Methods

### Study Design

The nationwide multicenter prospective study was implemented by the National Reference Center for Mycoses (NRCM, Institut Pasteur, France) between 1 April, 1997 and 1 July, 2001. The study was approved by the local ethical committee and reported to the French Ministry of Health (registration # DGS970089). All adults (i.e., ≥ 18 years of age), whether HIV infected or not, experiencing a first episode of culture-proven cryptococcosis, were eligible. Culture of blood, CSF, and urine were performed when the first evidence of cryptococcosis was found (i.e., positive culture from any body site, positive cryptococcal antigen testing, presence of encapsulated yeasts). Two weeks (Wk2) and three months (Mo3) after antifungal therapy was started, culture of initially infected body sites (i.e., those with positive culture) was requested. Patients' management regarding other investigations and therapeutic decisions was left to the physician in charge. Likewise, laboratory choices (cryptococcal antigen detection kit, medium, or duration of culture incubation) followed local practices.

A 16-page questionnaire (Text S1) was mailed upon receipt of the signed informed consent and anonymity was preserved. The information collected concerned epidemiological, clinical, biological, radiological, and mycological data at the time of antifungal therapy initiation (D0, baseline) as well as clinical and mycological status, treatment, and follow-up at Wk2 and Mo3. Any missing information or ambiguous answer was checked by phone with the physician or biologist in charge, and/or by careful review of the chart by at least one of us. All infecting isolates were collected and sent to the NRCM for serotyping [25].

### Definitions

A case was defined by isolation of C. neoformans from at least one body site. Classification of HIV-positive patients was based on the standard criteria established by the US Centers for Disease Control and Prevention in 1993 [26]. For each patient, extrapulmonary cryptococcosis was considered an AIDS-defining illness (sometimes revealing HIV infection) or not depending on the stage of HIV infection. Patients were classified according to their continent of birth (Europe, Africa, or others). Cases were classified as cryptococcal meningoencephalitis (assessed by *C. neoformans*-positive CSF culture, positive direct examination, and/or antigen testing [27]) or as extrameningeal cryptococcosis. Dissemination required that at least two noncontiguous body sites were infected. Abnormal neurology was defined by presence of seizures, abnormal mental status, and/or neurological defect [22,28,29]. Results of brain or thoracic imaging were reported by the clinician as normal or abnormal after local evaluation but without further description of the lesions. Initial therapy was recorded (systemic antifungal regimen administered for at least two consecutive days). Induction therapy defined the regimen that was used for at least five days during the acute phase of the disease, i.e., between baseline and the Wk2 evaluation. Clinical failure consisted of exacerbation of symptoms or death before evaluation, whereas clinical cure was defined by disappearance of all initial clinical abnormalities. Mycological outcome was evaluated only in patients for whom at least one body site was sampled at the time of workup (Wk2 or Mo3). Mycological failure meant that at least one of the cultured samples contained viable *C. neoformans;* mycological cure was defined as negative cultures for initially infected sites.

### Statistical Analysis

Means and standard deviations (SDs) are shown when distributions were confirmed normal; medians and interquartile ranges (IQRs) are reported otherwise. We compared baseline characteristics of groups by the χ^2^ test or Fisher exact test for categorical variables, and the t-test for continuous variables. Our principal outcomes were mycological failure at Wk2 and overall survival at Mo3. Thus, a difference was considered to be statistically significant after Bonferroni adjustment at *p* < 0.001 [30]. Other comparisons were exploratory analyses and we did not adjust the *p*-value.

For the multivariate analysis, we used logistic regression to determine factors associated with mycological failure at Wk2 in the total population, and to explain the lack of CSF sterilisation at Wk2 in HIV-positive patient with cryptococcal meningitis. Odds ratios (ORs) and their 95% confidence intervals (95% CIs) were determined by means of logistic regression analysis. Variables that were clinically relevant with *p* < 0.25 were entered simultaneously into the initial model. Variables were removed following a backward-stepwise selection procedure, leaving only variables with *p* < 0.05 in the final model. Moreover, interaction terms were explored on the basis of our final model. All variables retained were also tested by adding covariables (product of two variables) in the final logistic model. The statistical significance of interaction term was determined by *p*-value from the logistic regression.

Early deaths (before Wk2) were described and compared to late deaths (mortality rate). Finally, we analysed overall survival at Mo3. We estimated overall survival (cumulative survival probabilities and their 95% CIs) by the Kaplan-Meier method, and comparisons of survival between groups was performed by log-rank tests. Overall survival was measured from the date of diagnosis to the last follow-up or death from any cause.

**Figure 1:**
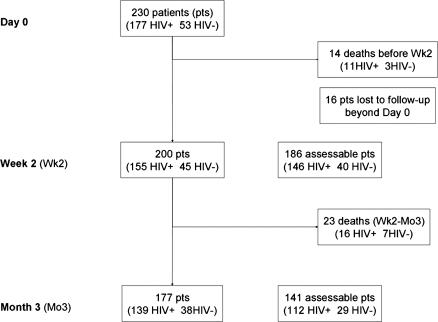
Number of Patients Alive, Assessable, and Dead at Each Study Point Data are from the French prospective multicentre CryptoA/D study. Pts, patients.

Each variable had a code corresponding to the absence of information. Thus, the analysis took into account only cases for which the corresponding parameter was available (lacking information was coded as missing value; Figure 1 shows the sample population at each study point). All variables were coded and analysed with Stata software v. 8.2 (Stata Statistical Software, http://www.stata.com).

## Results

### Demographic Characteristics of the Study Population

Over the 48 mo of study, 230 patients were enrolled in 77 medical centres throughout France, corresponding to 57% of the total number of cases reported to the ongoing nationwide surveillance program [31]. Heavier work load for the staff taking care of the patient and refusal to sign the consent accounted for most reasons of nonentry. There was no difference in the inclusion rate according to the year of study, the patient's gender or HIV status, or the presence of CSF, blood, urine, or skin involvement (Table S1).

The majority (142 [62%]) of the patients were HIV infected and male. Characteristics differed significantly between HIV-positive and -negative patients, with a higher proportion of patients born in Africa (59 [33.5%] versus 7 [3%], *p* = 0.005) and living in the Paris area (112 [63%] versus 21 [40%], *p* = 0.003), and a smaller proportion of patients in frequent contact with bird droppings (21/160 [13%] versus 16/48 [33%], *p* = 0.002) in HIV-positive compared to HIV-negative patients. Of note, more than half of the HIV-positive (57%) and -negative (51%) patients were or had been smokers.

#### HIV-positive patients.

Among the 177 HIV-positive patients, females were significantly younger (mean age in years ± SD: 36.3 ± 9.8 versus 39.9 ± 8.0, *p* = 0.027) and more frequently born in Africa (23 [66%] versus 36 [26%], *p* < 0.001) than males. There was no difference between male and female patients in the percentage of cryptococcosis defining AIDS (65%) including those revealing HIV infection (55%), diagnosed simultaneously with another opportunistic infection. CD4^+^ T lymphocytes cell counts/μl (mean ± SD = 45.1 ± 65.8), viral load (log_10_ copies/ml) (4.9 ± 1.0), and the proportion of patients treated by antiretroviral drugs at baseline (40%) were similar among genders (Table S2). In addition to the cellular immune defect related to HIV infection, other potentially predisposing conditions were found: pregnancy (two patients), infections with hepatitis B and C viruses (three and two patients, respectively) [32]. One patient was infected with HIV type 2.

#### HIV-negative patients.

Among the 53 HIV-negative patients, four groups were defined: group 1, solid organ transplantation (11 patients); group 2, malignancies (21 patients); group 3, various underlying disorders or treatments (12 patients); and group 4, no identified risk factors (nine patients). Transplanted organs were kidneys (eight cases), liver (two cases), and heart (one case). Among the patients with malignancies, three had solid tumours and 19 had haematological malignancies (seven lymphomas and 12 lymphoid leukaemias). In groups 1 and 2, additional potential risk factors were identified in 15 patients (diabetes mellitus, chronic renal failure, tuberculosis, hepatitis B virus-related cirrhosis, chronic hepatitis C). In group 3, risk factors associated with potential or demonstrated immunosuppression were those described in other studies (diabetes mellitus, cirrhosis, sarcoidosis, idiopathic CD4^+^ T lymphocytopenia, hypogammaglobulinaemia, corticosteroid therapy) [20,21,28]. Among the nine patients without any identified risk factors, five had experienced a recent injury evoking primary cutaneous cryptococcosis [33]. Recent (<3 mo) or current immunosuppressive regimens including corticosteroids were prescribed to 62% of all patients (none in group 4).

### Baseline Clinical and Radiological Characteristics of Patients with Incident Cryptococcosis

Baseline clinical characteristics and first symptoms of cryptococcosis differed according to HIV status (Tables 1 and S3). Intracranial pressure was not monitored routinely. Overall, the first notified symptom was in order of frequency: headache (75 [34%]), fever (50 [23%]), abnormal mental status, skin lesions, and cough (19–20 each [9%]). Mean time between onset of symptoms and hospitalisation was significantly longer for HIV-negative than HIV-positive patients with meningoencephalitis (mean ± SD in weeks, 6.0 ± 7.4 versus 3.3 ± 3.3, *p* = 0.0019).

**Table 1:**
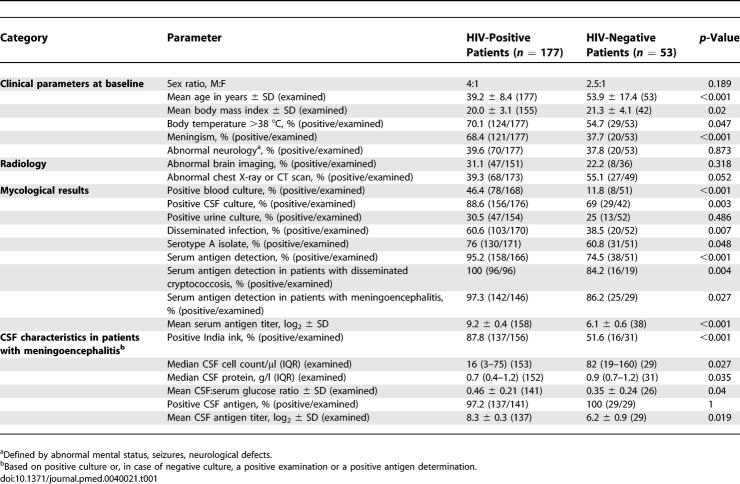
Baseline Clinical, Radiological, and Mycological Characteristics in 230 Adult Patients with Culture-Confirmed Cryptococcosis According to HIV Serostatus

Abnormal neurology (abnormal mental status [62 (33%)], motor or cranial nerves palsies [29 (15%)], seizures [18 (10%)]) was observed in almost half (86 [46%]) of patients with cryptococcal meningoencephalitis without a difference according to HIV status. Abnormal thoracic images were more frequent among HIV-negative patients, but nonrecorded unrelated conditions could account for these chest X-ray abnormalities, preventing further analysis.

### Baseline Mycological and CSF Characteristics of Patients with Incident Cryptococcosis

At least two body sites were cultured in 222 [97%] of the patients. Cryptococcal meningoencephalitis was mostly diagnosed by culture except in one HIV-positive patient (positive antigen) and two HIV-negative patients (one positive antigen and one positive India ink).

CSF characteristics differed between HIV-positive and -negative patients with meningoencephalitis (Tables 1 and S3). Four HIV-positive patients with positive CSF culture, including two with positive India ink test, had negative antigen detection in CSF. Three of the 19 HIV-negative patients with disseminated cryptococcosis and two of six HIV-negative patients with fungaemia had negative serum antigen detection compared to none of the HIV-positive patients in the same situation (*n* = 96 and 74, *p* = 0.028 and 0.005, respectively). Among patients with extrameningeal cryptococcosis, 20% had negative antigen detection without a difference according to HIV status. Finally, a discrepancy in antigen detection between serum and CSF (negative in serum and positive in CSF) was seen in six cases (two HIV-positive and four HIV-negative patients, all with positive CSF culture and only one with disseminated infection). Overall, serum antigen titres were significantly higher in patients with disseminated infection or fungaemia than in those without (*p* < 0.001). A threshold of antigen titer (≥1:512) was chosen for most of the analyses, since the proportion of samples with high antigen titres was not significantly different according to the commercial kit used for titration (unpublished data).

### Differences of Baseline Characteristics According to Sex in HIV-Positive Patients

Fungaemia was significantly more frequent among male than among female patients, as were positive urine cultures and disseminated infections (Table 2). In patients with meningoencephalitis, characteristics of the CSF were similar between genders. Serum but not CSF antigen titres were significantly higher in males than in females. The time interval between hospitalisation and onset of antifungal therapy tended to be longer for female than for male patients. The only significant difference among HIV-negative patients was a lower CSF antigen titre recorded in female versus male patients (Table S4).

**Table 2:**
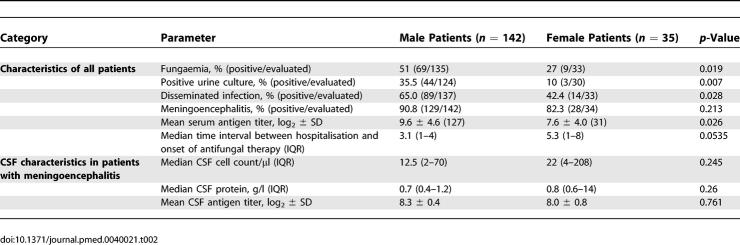
Characteristics of Cryptococcosis that Significantly Differed between Sexes in 177 HIV-Infected Patients with Cryptococcosis

### Differences According to the Infecting Variety/Serotype of C. neoformans

The influence of the infecting serotype on the clinical presentation and outcome differed in HIV-positive and -negative patients (Table 3). Similar patterns were seen in both populations regarding the continent of origin, the proportion of smokers, proportion of severe hyponatraemia, and the delay in antifungal drug prescriptions while trends were divergent for others (serum and CSF antigen titres, proportion of meningoencephalitis) or seen only in HIV-negative patients (higher proportion of death from cryptococcosis).

**Table 3:**
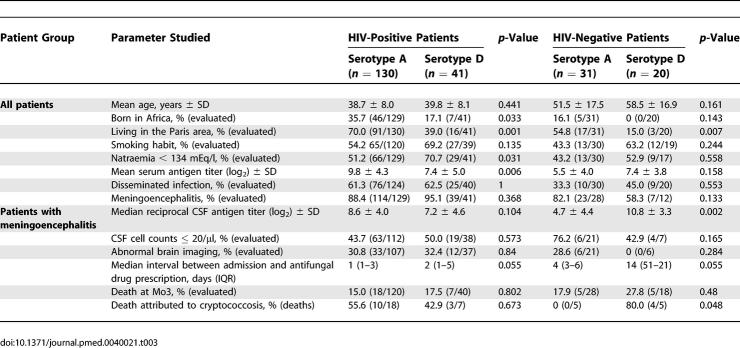
Comparisons of Infections Due to C. neoformans Serotype A (var. *grubii*) and Serotype D (var. *neoformans*) According to HIV Status

### Outcome Two Weeks after Onset of Antifungal Treatment

The most frequently prescribed antifungal therapy during the induction phase was the combination of amphotericin B and flucytosine in HIV-positive patients (91/176 [52%] versus 15/53 [28%] in HIV-negative patients, *p* = 0.003) while the majority (31/53 [58%]) of HIV-negative patients received monotherapy with amphotericin B or fluconazole. Use of flucytosine during induction therapy was not altered by HIV status in patients with meningoencephalitis even though it was less frequent in HIV-seronegative patients with haematological malignancies (91/157 [58%] in HIV-positive patients; 50%–66% in groups 1 (4/8), 3 (4/7), and 4 (2/3) of HIV-negative patients; and 4/13 [31%] in group 2; *p* = 0.425). To avoid the bias in daily dosage linked to prescription habits or underlying diseases we analysed therapeutic efficacy in terms of cumulative doses (= daily dosage × duration). Mean durations of induction therapy were not significantly different according to HIV serostatus (12.3 ± 4.3 d versus 13.9 ± 4.7 d for flucytosine, *p* = 0.202, and 13.4 ± 3.9 d versus 12.7 ± 3.3 d for amphotericin B, *p* = 0.338, respectively, in HIV-positive and -negative patients).

Two weeks after the diagnosis of cryptococcosis, outcomes were available for 214 patients, of whom 14 died (Figure 1). These early deaths (14/37 [38%] of all deaths) were recorded in three HIV-negative and 11 HIV-positive patients, including two without meningoencephalitis. They were more often attributed to cryptococcosis than were deaths occurring later (12/14 [86%] versus 7/23 [30%], *p* = 0.002). Early deaths were also more frequent among patients with abnormal neurology (10/86 [12%] versus 4/128 [3%], *p* = 0.021), abnormal brain imaging (6/60 [12%] versus 2/125 [2%], *p* = 0.007), abnormal thoracic imaging (10/89 [11%] versus 4/119 [3%], *p* = 0.047), and hyponatraemia (12/115 [10%] versus 2/95 [2%], *p* = 0.023). The presence of meningoencephalitis, HIV status, or sex did not influence the proportions.

Of 200 patients (92%) that remained alive, 186 (93%) had body fluids sampled for culture (Figure 1). Among the patients alive at Wk2, clinical cure was obtained in 115 of 200 patients (57.5%) with no difference according to HIV status or sex. However, clinical cure was less frequently recorded among patients with abnormal neurology (31/76 [41%] versus 84/124 [68%], *p* < 0.001) or meningoencephalitis (90/168 [54%] versus 19/24 [78%], *p* = 0.026) at baseline than among others.

Control of sterilisation of the initially infected site was performed for 150/178 [84%] of CSF, 53/79 [67%] of blood, and 44/52 [85%] of urine samples. Among those considered clinically cured, 29/105 [28%] still had one body site infected, while among those who were considered clinical failures, 43/81 [53%] were sterilised (*p* = 0.009). Encapsulated yeasts were still seen in 97/148 [65%] of the CSF examined (87/128 [70%] and 10/23 [43%] of the HIV-positive and -negative patients, respectively, *p* = 0.030). CSF culture was still positive in 61/151 [40%] of the patients with initial meningoencephalitis, with no difference according to HIV status. Overall, sterilisation was not achieved in 67/186 [36%] of the patients after two weeks of antifungal therapy.

#### Factors associated with mycological failure after two weeks of antifungal therapy.

We analysed, in the 186 patients assessable at the Wk2 workup, the factors associated with mycological failure whatever the initial presentation (presence or absence of meningoencephalitis) and the HIV status (Table 4). In the absence of flucytosine for induction therapy, mycological failure was recorded in no solid organ transplant recipients, 1/4 and 3/12 [25%] of seronegative patients with no risk factor (group 4) or haematological malignancies (group 2), respectively, and in 2/4 [50%] and 31/68 [54%] of patients with miscellaneous risk factors (group 3) or HIV infection, respectively (*p* = 0.075). In the multivariate analysis (178 patients), presence of initial dissemination (OR, 2.4 [95% CI, 1.2–4.9], *p* = 0.015), high serum antigen titer (OR, 2.6 [95% CI, 1.3–5.4], *p* = 0.008), and lack of flucytosine during induction therapy (OR, 3.8 [95% CI, 1.9–7.8], *p* < 0.001) were independently associated with mycological failure at Wk2 (interaction terms among the three factors were not significant).

**Table 4:**
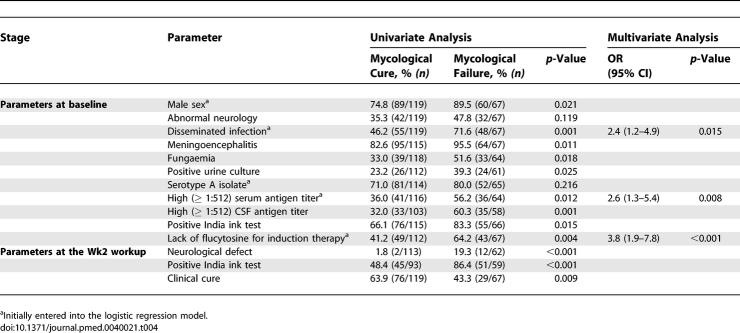
Parameters Associated with Mycological Failure at the Wk2 Workup in 186 HIV-Positive and -Negative patients with Cryptococcosis (All Cases): Univariate and Multivariate Analyses

#### Factors associated with lack of CSF sterilisation in HIV-positive patients with cryptococcal meningoencephalitis.

HIV-related parameters (CD4^+^ cell count, viral load) had no influence on the percentage of CSF sterilisation (Table 5). The mean cumulative dose of flucytosine (and the duration but not the daily dosage, unpublished data) was lower in cases of positive compared to negative CSF Wk2 cultures (mean dose in grams ± SD = 69 ± 49 versus 94 ± 41, *p* = 0.015), whereas there was no difference in the mean cumulative dose of fluconazole or amphotericin B. In a multivariate analysis involving 111 patients, an infection by serotype A (OR, 5.6 [95% CI, 1.6–19.8], *p* = 0.008), a high CSF antigen titer at baseline (OR, 14.1 [95% CI, 3.0–67.1], *p* = 0.001) and the lack of flucytosine prescription during induction therapy (OR, 24.4 [95% CI, 4.8–123.5], *p* < 0.001) were independently associated with lack of CSF sterilisation at Wk2 (interaction terms among the three factors were not significant).

**Table 5:**
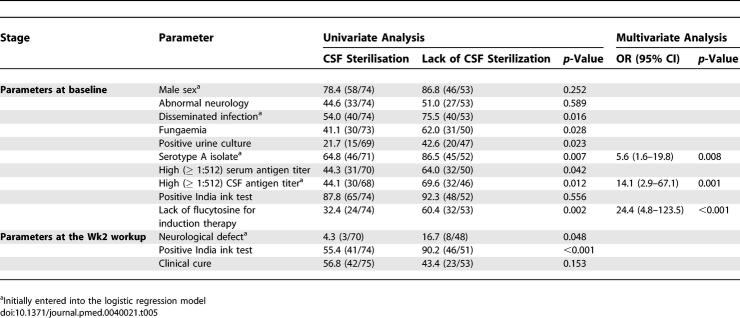
Parameters Associated with Lack of CSF Sterilisation in 127 HIV-Positive Patients with Cryptococcal Meningoencephalitis: Univariate and Multivariate Analyses

### Factors Associated with Death within Three Months of Diagnosis of Cryptococcosis

At Mo3 (Figure 1), 177 patients remained alive, and neurological sequelae were observed in seven HIV-positive patients. Of the 177 survivors, 142 (80%) had a mycological evaluation at Mo3. Three HIV-positive patients had still viable yeasts in the CSF and one HIV-negative patient had a positive bronchoalveolar lavage culture.

Overall, 37 patients (27 HIV-positive and ten HIV-negative) died during follow-up in relation with cryptococcosis (19 patients, including four HIV-negative patients) or their underlying disease (18 patients, including six HIV-negative patients). None of the seven HIV-negative immunocompetent patients (group 4) died, including the three with meningoencephalitis.

**Figure 2:**
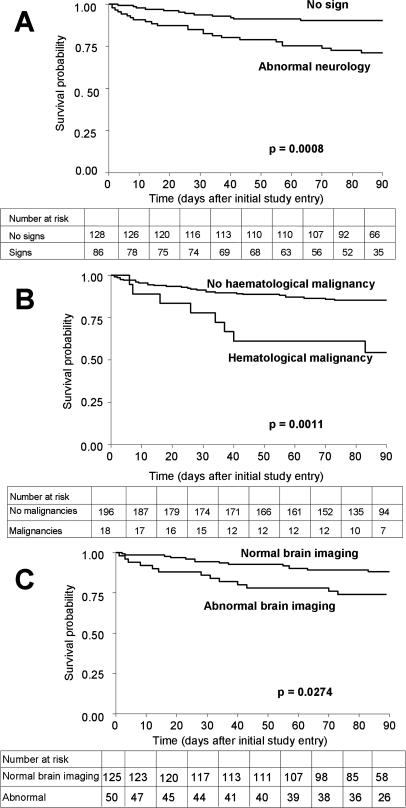
Overall Survival at Three Months after the Diagnosis of Cryptococcosis (A) Patients presenting with and without abnormal neurology at baseline. (B) Patients with and without haematological malignancies. (C) Patients presenting with and without abnormal brain imaging at baseline (data missing for 39 patients).

The survival probability at Mo3 was thus lower in patients with abnormal neurology at baseline (survival probability 0.71 [95% CI, 0.60–0.80]) compared to those without (0.90 [95% CI, 0.84–0.94], *p* = 0.0008), in patients with haematological malignancies (0.54 [95% CI, 0.29–0.74]) compared to those without (0.85 [95% CI, 0.79–0.90], *p* = 0.011), and in patients with abnormal brain imaging at baseline (0.74 [95% CI, 0.53–0.83]) compared to those with normal brain imaging (0.88 [95% CI, 0.81–0.93], *p* = 0.0274) (Figure 2). Abnormal neurology and abnormal brain imaging still influenced survival probability when patients with concomitant or past cerebral toxoplasmosis were excluded (unpublished data).

## Discussion

We analysed in a prospective study the determinants of clinical presentation and outcome in patients with incident cryptococcosis in France, 1997–2001. Based on this population, cryptococcosis was more severe in men, in HIV-positive patients, and patients infected with serotype A C. neoformans. Factors independently associated with mycological failure at Wk2, regardless of the HIV status, were: initial dissemination, high serum antigen titer at baseline, and lack of flucytosine during induction therapy. The Mo3 survival was lower in patients with abnormal neurology or abnormal brain imaging at baseline, and in those with haematological malignancy.

As this was an observational study, there are limitations to the data available for analysis despite a low rate of patients lost to follow-up. Principal among these limitations is the potential for selection of the patients. Although we verified that patients enrolled in the CryptoA/D study and those notified through the nationwide epidemiological survey did not differ according to type of underling disease, sex, and cryptococcosis presentation (especially the proportion of extrameningeal infection), other patients could have been left out of both studies. Our study reflects the situation in a European country where serotypes A and D are present in the environment. Other data could be obtained in a different environment especially in countries where C. gattii exists. Handling of the biological samples was not centralised, which could introduce variations in the yield of positive cultures due to variation in laboratory practices. Finally, the question of multiple comparisons adjustment is a subject of debate in epidemiological research [30,34], arising because of the increased risk of type I errors (findings of false significance) when numerous hypotheses are tested simultaneously at set *p*-values. We thus limited the adjustment to the main outcomes and engaged other comparisons to present exploratory but relevant results [35].

CSF cultures were more frequently positive for C. neoformans in HIV-positive than in HIV-negative patients [2,31]. There was more pronounced cryptococcosis-associated CSF inflammation in HIV-negative patients than in HIV-positive patients. This corroborates previous studies comparing inflammatory cytokine and mediator levels in CSF during cryptococcosis in humans [36] or demonstrating the role of inflammatory cytokines during cryptococcosis [37–39]. Fungaemia is probably a critical step in the development and persistence of meningoencephalitis [40] as further evidenced by a higher percentage of fungaemia paralleling the higher percentage of meningoencephalitis at baseline and of CSF nonsterilisation at Wk2 in HIV-positive patients. It even suggests that C. neoformans persistently circulating in blood may contribute to subsequent reinfection of the central nervous system, an hypothesis supported by data obtained in the early stage of infection in a mouse model of disseminated cryptococcosis [41].

We found a significant difference in the proportion of males and females in HIV-positive and -negative patients with cryptococcosis (the male:female ratio among patients with AIDS in France was then 2.8:1 [42]). In addition, there was a sex difference in terms of disease characteristics at baseline. A higher production of inflammatory cytokines was measured in female versus male mice associated with a nonsignificant trend toward a lower fungal burden in females [43]. Overall, these data suggest the influence of sex hormones—possibly through inflammatory mediators—in the control of cryptococcosis and not only in the susceptibility to infection.

In clinical practice, cryptococcal antigen detection and titration are used to evaluate dissemination, severity of disease, and response to treatment. Negative antigen detection in serum does not rule out dissemination even in HIV-negative patients (three cases in this study). Likewise, negative antigen detection in CSF, although rare, is not incompatible with true meningoencephalitis [21]. We showed that antigen titres were significantly higher in all situations associated with evidence of dissemination. High CSF antigen titres have been associated with lack of CSF sterilisation [44,45] or death [22,44,46] in HIV-positive patients with meningoencephalitis. An association between high serum antigen titres and early outcome has not been demonstrated in HIV-negative patients or extrameningeal cryptococcosis since a study published with the first agglutination test [28]. A threshold of 1:512 or higher should thus help monitor patients with cryptococcosis whatever their HIV status. A serum antigen titer 1:512 or higher during follow-up was also identified as an independent factor of cryptococcosis relapse in a study of the long-term outcome of cryptococcosis in patients with AIDS [47].

The Wk2 workup is now considered a major milestone in the management of cryptococcal meningitis. Indeed, the recommended treatment is a combination of amphotericin B (0.7 mg/kg/d) and flucytosine (100 mg/kg/d) for two weeks, followed by fluconazole at a dosage of 400 mg/d or higher for ten weeks [18,22,24]. Flucytosine prescription has been associated with toxicity [19,24,48], but lower dosages and monitoring of plasma drug levels can prevent this problem. Combination therapy is associated with lower rates of subsequent relapse during maintenance therapy [23] and sterilisation of CSF as determined by culture [19]. Recent data obtained in HIV-positive patients in Thailand showed also that clearance of cryptococci from the CSF was significantly faster with amphotericin B–flucytosine compared to amphotericin B alone or other combination therapies [49]. Here, the lack of flucytosine—and not only the lack of combination therapy with amphotericin B and flucytosine—during the induction phase was independently associated with lack of CSF sterilisation (HIV-positive patients) or mycological failure (entire population) at Wk2. We showed in vitro and in a murine model of cryptococcosis that synergy can be achieved with amphotericin B–flucytosine combination therapy even if the isolate is resistant to flucytosine in vitro [50–52]. Thus, together with data previously published in HIV-positive patients showing that early mycological failure can be associated with death [45], our results support the prescription of flucytosine for induction treatment of cryptococcosis in all situations in which a high fungal burden is suspected based on antigen titration or cultures, regardless of HIV status.

We observed an overall mortality rate in patients with cryptococcosis of 6.5% in the first two weeks and 11.5% in the next ten weeks, comparable to other reports [22,45] but higher than the most recent randomized trial of the Mycoses Study Group [24]. The higher mortality rate observed in our study can be explained by a high proportion of severe cases as well as by the lack of systematic management of intracranial pressure in France. High intracranial pressure has been associated with poor prognosis [44], and failure to control it has been linked to neurological injuries [53]. Factors predictive of deaths within three months after the diagnosis were underlying haematological malignancy, presence at baseline of abnormal neurology, or abnormal brain imaging. Despite the apparent contraction, survival was reported lower in HIV-negative compared to HIV-positive patients even before the introduction of HAART [54], and the mortality rate was even higher in HIV-negative patients with haematological malignancies [20,21]. Abnormal neurology has already been associated with a poor prognosis in several studies [28,29]. Abnormal brain imaging at baseline was uncovered here as a parameter associated with death from cryptococcosis. Only small series of computerized tomographies or magnetic resonance imaging of the brain have been published so far, and none has clearly addressed the relationship between cerebral lesions and prognosis [1]. Thus, brain lesions specifically associated with a poor prognosis are currently being analysed on a subset of patients with meningoencephalitis based on blinded review of the brain images.

Finally, despite data indicating that serotypes A and D differ in terms of host infected, clinical presentation, and even early outcome, differences are far less striking than those reported between C. neoformans and C. gattii [15–17], thus supporting the current concept of two varieties of the same species rather than two species [55]. Despite additional differences between serotypes A and D in terms of susceptibility to amphotericin B and fluconazole but not flucytosine, the similar long-term outcome may be explained by the lack of correlation between in vitro and in vivo results [56] or other still uncovered factors.

Despite the decline in the incidence of cryptococcosis, mortality rate remains about 15%–20% at Mo3 in HIV-negative and HIV-positive patients in countries where HAART is available [47] and much higher in some countries of Africa and Southeast Asia [5,57,58], thus calling for improvement of therapeutic management. Patients who should benefit from a two-week induction therapy with amphotericin B and flucytosine are those with meningoencephalitis or severe pneumonia, regardless of HIV status, as already proposed in the Infectious Diseases Society of America guidelines [18]. We suggest adding to the current recommendations patients with high fungal burden regardless of HIV status, i.e., those with serum antigen titer 1:512 or higher, or those with fungaemia or disseminated infection (defined by two noncontiguous infected body sites including urine). Our current analysis does not allow us to alter the recommendations concerning the induction therapy for other clinical presentations (400 mg/d of fluconazole) or for the consolidation phase (fluconazole 400 mg/d for ten weeks). We strongly advocate, however, that evidence of cryptococcosis—based on positive antigen detection and/or presence of encapsulated yeasts at direct examination/histology and/or isolation of C. neoformans from any body site—be immediately followed by sampling and culture of CSF, blood, and urine, and by serum antigen titration in order to evaluate fungal burden and optimize induction treatment.

## Supporting Information

Table S1 Comparison of Patients' Characteristics According to Their Enrolment in the CryptoA/D Study versus Notification to the National Reference Center through Ongoing Surveillance Program on Cryptococcosis in France (Survey) during the Years 1997–2001 (21 KB XLS)

Table S2 Additional Comparison of HIV Infection According to Sex in 177 HIV-Positive Patients with Cryptococcosis (20 KB XLS)

Table S3 Characteristics of Cryptococcosis at Baseline among HIV-Positive and -Negative Patients (20 KB XLS)

Table S4 Characteristics of Cryptococcosis According to Sex among the 53 HIV-Negative Patients (21 KB XLS)

Text S1 CryptoA/D Study Questionnaire (655 KB PDF)

Alternative Language Abstract S1 Translation of the Abstract into French by F. Dromer (39 KB DOC)

## Abbreviations

CI: confidence interval
CSF: cerebrospinal fluid
HAART: highly active antiretroviral therapy
IQR: interquartile range
Mo3: three months after onset of antifungal therapy
OR: odds ratio
SD: standard deviation
Wk2: two weeks after onset of antifungal therapy
