# Supplementary material for: Determinants of Disease Presentation and Outcome during Cryptococcosis: The CryptoA/D Study
Source: PLoS Med. 2007 Feb 6;4(2):e21. doi: 10.1371/journal.pmed.0040021 (PMC1808080; doi:10.1371/journal.pmed.0040021)
Supplement: Text S1 — (655 KB PDF) [file pmed.0040021.sd001.pdf]

CRYPTOA/D STUDY

INVESTIGATORS

Françoise Dromer, principal investigator
Olivier Lortholary
French Cryptococcosis Study Group

SUPPORT

Institut Pasteur (promotor)
Société Française de Mycologie Médicale
Société Nationale Française de Médecine Interne
Société de Pathologie Infectieuse de Langue Française

National Reference Center for Mycoses & Antifungals
Institut Pasteur, 25, rue du Dr. Roux, 75724 Paris cedex 15
Phone : 33 1 40 61 36 90
FAX : 33 1 45 68 84 20
E-mail : dromer@pasteur.fr

CRYPTO A/D STUDY

CODE

QUESTIONNAIRE

PATIENT # |__|__|__|__|__|__|
(assigned by NRCM)

CLINICIAN'S NAME: ....

HOSPITAL & WARD DESIGNATION :

....

....

ADDRESS ....

CITY : .... ZIP |__|__|__|__|__|

PHONE : .... FAX : ....

MYCOLOGIST'S NAME : ....

HÔSPITAL & LABORATORY : ....

....

ADDRESS ....

CITY : .... ZIP |__|__|__|__|__|

PHONE : .... FAX : ....

DATES

INCLUSION (**Day0** of treatment) : d/m/y |__|__| |__|__| |__|__|

FOLLOW-UP WEEK2 (WK2) : Expected date |__|__| |__|__| |__|__|

Real date |__|__| |__|__| |__|__|

FOLLOW-UP WEEK 12 (MO3) : Expected date |__|__| |__|__| |__|__|

Real date |__|__| |__|__| |__|__|

BASELINE

Enrollement date (Day0) is the first day of antifungal therapy

I. SOCIO-DEMOGRAPHIC CHARACTERISTICS

CODE

1. Date of birth: |__|__| day |__|__| month |__|__| year ☐☐☐☐☐☐

2. Gender: ☐ male ☐ female ☐

3. Continent of origin : ☐ Europe ☐ North Africa ☐ Central Africa ☐ Caribbean islands ☐ Asia ☐ Others ……………………… ☐

4. Department of birth (country if born in a foreign country) : .... ☐☐☐

5. Arrival in France : |__|__| month |__|__| year ☐☐☐☐

6. Zip code of the living area: |__|__| ☐☐

7. Arrival in that area : |__|__| month |__|__| year ☐☐☐☐

8. Regions of France visited by the patient : ☐

	NEVER	OCCASIONNALY (< 2months)	PROLONGED stay (≥ 6 months)	year of the last visit	CODE
SouthWest :	☐	☐	☐	|__|__|	☐☐☐
West :	☐	☐	☐	|__|__|	☐☐☐
East :	☐	☐	☐	|__|__|	☐☐☐
Central part :	☐	☐	☐	|__|__|	☐☐☐
North :	☐	☐	☐	|__|__|	☐☐☐
South-East :	☐	☐	☐	|__|__|	☐☐☐
Alpes :	☐	☐	☐	|__|__|	☐☐☐
Paris area:	☐	☐	☐	|__|__|	☐☐☐

Details if necessary: ……………………………………………………………………………

……………………………………………………………………………………………………

……………………………………………………………………………………………………

** articles 26, 27, 34 et 40 de la loi n°78-17 du 6/01/1978 relative à l'informatique, aux fichiers et aux libertés*

CODE

9. Visit to foreign country **including during childhood**

	NEVER	OCCASIONNALLY (< 2months)	PROLONGED Visit (≥ 6 months)	year of the last visit
North Africa :	☐	☐	☐	└─┴─┘
Central Africa :	☐	☐	☐	└─┴─┘
Caribbean islands :	☐	☐	☐	└─┴─┘
North America :	☐	☐	☐	└─┴─┘
South America :	☐	☐	☐	└─┴─┘
Asia :	☐	☐	☐	└─┴─┘

DETAILS if necessary : ....

....

....

PROFESSION

10. Current occupation : ....

11. Building worker: ☐ Yes ☐ No

12. Professional contact with dusts : ☐ Yes ☐ No

13. Frequent contacts with birds/poultry: ☐ Yes ☐ No

MODE DE VIE

14. Smoking habit : ☐ current ☐ past ☐ never

15. if smoker, boxes/year : ☐ 1-9 ☐ 10-19 ☐ ≥ 20

16. Drug addiction

intravenous : ☐ current ☐ past ☐ never

inhalation : ☐ current ☐ past ☐ never

17. Duration: ☐ < 1 month ☐ 1 month-<1year ☐ 1-5 years ☐ > 5years

List drugs : ....

18. Alcoholism : ☐ current ☐ past ☐ never

** articles 26, 27, 34 et 40 de la loi n°78-17 du 6/01/1978 relative à l'informatique, aux fichiers et aux libertés*

II. PREDISPOSING FACTORS

IIA. If the patient is HIV-infected

CODE

19. Date of HIV-seropositivity : |__|__| month |__|__| year

20. Date of AIDS |__|__| month |__|__| year

21. Disease(s) that allowed definition of AIDS stage: ………………………………

22. Route of HIV contamination:

☐ homo/bisexual ☐ drug addiction ☐ heterosexual ☐ others ………..

23. CD4 at the time of cryptococcosis diagnosis: ………../ mm^3 ……%

24. Viral load (copy number, technique): ……………………………………

25. Antiretroviral treatment at the time of cryptococcosis diagnosis

☐ 2 drugs ☐ 3 drugs ☐ 4 drugs

Date of 1st prescription of antiviral Rx: |__|__| month |__|__| year

25 bis. Treatment including protease inhibitor(s) ☐ yes ☐ no

Date of 1st prescription of the protease inhibitor: |__|__| |__|__| |__|__|

26. Malignancy : ☐ lymphoma ☐ Kaposi ☐ None

27. Number of opportunistic nonfungal infections before cryptococcosis: ☐

Please give date and diagnosis of all the OI: ………………………………………......................

………………………………………………………………………………………

B|____| P|____| V|____|

28. Previous history of prostatitis: ☐ Yes ☐ No

IIB. PREVIOUS HISTORY OF MYCOSES

29. Oropharyngeal candidiasis : ☐ none ☐ < 5 ☐ 5-10 ☐ > 10 episodes

30. Candida oesophagitis : ☐ none ☐ < 5 ☐ 5-10 ☐ > 10 episodes

31. Other mycoses: ☐ none ☐ histoplasmosis ☐ aspergillosis

32. Prior treatment with fluconazole:

☐ never ☐ current ☐ stopped on |__|__| |__|__|

Cumulative dose ☐ < 2 g ☐ 2-10 g ☐ > 10g

33. Prior treatment with itraconazole:

☐ Never ☐ current ☐ stropped on |__|__| |__|__|

Cumulative dose ☐ < 2 g ☐ 2-10 g ☐ > 10g

34. Prior treatment with intravenous amphotericin B:

☐ Never ☐ current ☐ stopped on |__|__| |__|__|

Cumulative dose ☐ < 0.5 g ☐ 0.5 - 1 g ☐ > 1 g

** articles 26, 27, 34 et 40 de la loi n°78-17 du 6/01/1978 relative à l'informatique, aux fichiers et aux libertés*

IIC. OTHER FACTORS ABLE TO PREDISPOSE TO CRYPTOCOCCOSIS

to be filled even if the patient is HIV-negative

CODE

35. HIV serology: ☐ negative ☐ not done ☐

36. CD4 at the time of cryptococcosis diagnosis : ………../ mm^3 ; ………% ☐☐☐
(importance even for HIV negative patients)

37. Solid tumor : ☐ **Yes**, in 19 |__|__| ☐ **No** ☐☐☐

Diagnosis ……………………………………………………………………………… |______|

38. Haematological malignancy: ☐ **Yes, in** 19 |__|__| ☐ **No** ☐☐☐

Diagnosis …………………………………………… ……………………………… |______|

39. Organ transplantation : ☐ **Yes, in** 19 |__|__| ☐ **No** ☐☐☐

Details: ……………………………………… |______|

40. Other diseases ☐ Yes ☐ No ☐
(you can check several boxes)

41. Diabetes mellitus : ☐ Yes ☐ No ☐

42. Chronic renal insufficiency: ☐ Yes ☐ No ☐

43. Cirrhosis : ☐ oui ☐ Yes ☐

44. Sarcoidosis : ☐ Yes ☐ No ☐

45. Idiopathic CD4 lymphocytopenia : ☐ Yes ☐ No ☐
(please go back to questions 27, 28 et §IIB)

46. Others (details) : ……………………………………………………………… ☐

47. Corticotherapy : ☐ Current ☐ stopped since **19**…… ☐ No ☐☐☐
(≥0,5 mg/kg/d > 8d)

48. Chemotherapy : ☐ Current ☐ stopped since **19**…… ☐ No ☐☐☐

49. Immunosuppressive drugs : ☐ current ☐ stopped since **19**…… ☐ No ☐☐☐

if yes, please detail……………………………………………………………………

50. In case of cutaneous lesions

Prior history of trauma? ☐ Yes ☐ No ☐

If yes : ☐ injury ☐ puncture ☐ insect bite or ………………… |______|

Localisation compared to the cutaneous lesion: ☐ identical ☐ different ☐

Date of the trauma: |__|__| day |__|__| month |__|__| year ☐☐☐☐☐☐

GROUP ☐

** articles 26, 27, 34 et 40 de la loi n°78-17 du 6/01/1978 relative à l'informatique, aux fichiers et aux libertés*

III. CLINICAL FEATURES ON DAY 0

CODES

DATE D0 |__|__| |__|__| |__|__|

IIIA.CLINICAL SIGNS

51. Systolic arterial pressure: |__|__|__| mm Hg

52. Pulse: |__|__|__| /mn

53. Fever (temperature ≥ 38°C) : ☐ Yes ☐ No

54. Meningism: ☐ Yes ☐ No

55a. Abnormal mental status (obnubilation or coma) : ☐ Yes ☐ No

55b. Seizures : ☐ Yes ☐ No

56. Cranial nerve defect : ☐ Yes ☐ No

57. Motor defect : ☐ Yes ☐ No

58. Eye exam : ☐ normal ☐ papilla edema ☐ retinitis ☐ not done

59. CSF opening pressure …………… cm

60. Acute dyspnea : ☐ Yes ☐ No

61. Cough : ☐ Yes ☐ No

62. Cutaneous lesions : ☐ Yes ☐ No

☐ papules ☐ cellulitis ☐ ulcerations |________| others

number of lesions : ☐ 1 ☐ ≥ 2 ☐ ≥ 10

63. First symptom to appear (among items 53-57 & 60-62) : ……………………

Time between onset and hospitalisation (in weeks) : |__|__|

64. Other information :

Urinary catheter : ☐ Yes ☐ No

Central catheter : ☐ Yes ☐ No

Tracheal intubation : ☐ Yes ☐ No

** articles 26, 27, 34 et 40 de la loi n°78-17 du 6/01/1978 relative à l'informatique, aux fichiers et aux libertés*

IIIB. EXPLORATIONS (*sampling* DAY-2 TO DAY+2)

ATTENTION : **MANDATORY CULTURES ARE UNDERLINED & IN BOLD**
SAMPLES TO BE STORED ARE INDICATED by (☒)

Blood biology

CODE

65. Leucocyte number : ☐☐☐☐☐ /mm^3 ☐☐☐☐☐

PMN : ……………… % ☐☐

Lymphocytes : ……………… % ☐☐

66. Natremia : ☐☐☐ mEq ☐☐☐

67. Glycemia : ☐☐☐ mmols/l ☐☐☐

Pulmonary investigations

68. Chest X-ray**:** ☐ normal ☐ abnormal ☐ not done ☐

Si abnormal, please check the corresponding boxe(s):

Alveolar condensation	☐ Yes	☐ No	
Interstitial lesions	☐ Yes	☐ No	
Nodules < 2 cm	☐ Yes	☐ No	
Mass > 2 cm	☐ Yes	☐ No	
Cavity	☐ Yes	☐ No	
Mediastinal lymph nodes	☐ Yes	☐ No	
Hilar lymph nodes	☐ Yes	☐ No	
Pleuritis	☐ Yes	☐ No	

Others : ………………………………………………

69. Thoracic CT-scan: ☐ normal ☐ abnormal ☐ not done ☐

70. Lung fibroscopy ☐ Yes ☐ No ☐

Lung biopsy: ☐ Yes ☐ No ☐

* **Merci de joindre la photocopie du compte-rendu**

Cerebral investigatons

71. Brain CT-scan: ☐ normal ☐ abnormal ☐ not done ☐

72. Brain MRI : ☐ normal ☐ abnormal ☐ not done ☐

IV. MYCOLOGICAL INVESTIGATIONS

ATTENTION : **MANDATORY CULTURES (bold & underlined)**
SAMPLES TO STORE (☒)

BLOOD ☒ **(≈ 5 ml plasma to be stored frozen)**

CODE

73. Cryptococcal antigen detection : ☐ positive ☐ negative
Please, provide brand's name: ……………………… and titer : ………..

BLOOD CULTURE

date : |__|__| day |__|__| month

74. Culture : ☐ positive ☐ negative
Technique : ………………… Medium………………………
Other results : ………………………………

CEREBROSPINAL FLUID ☒ ***(store 1 ml of supernatant frozen = 25 drops)*** :

date : |__|__| day |__|__| month

75. Cells (number & type /mm^3) : ....
76. CSF proteins : ....g / l.
77. CSF glucose : .... mmoles/l
78. India ink staining : ☐ positive ☐ negative
79. CULTURE : ☐ positive ☐ negative
80. CSF cryptococcal antigen : ☐ positive ☐ negative ☐ not done
Titer : …………………………………

URINES ☒ ***(store 5 ml of supernatant frozen)***

Date of sampling: |__|__| day |__|__| month

81. Capsulated yeasts at direct examination : ☐ Yes ☐ No ☐ ND
82. CULTURE of *C. neoformans* : ☐ positive ☐ negative
83. Culture other than *C. neoformans* : ☐ oui ☐ non
if yes, details ☐ Bacteria : ……………………………………
☐ *Candida* sp. : ………………………………
Cells/ml ☐ < 10^4/ml ☐ ≥ 10^4/ml

** articles 26, 27, 34 et 40 de la loi n°78-17 du 6/01/1978 relative à l'informatique, aux fichiers et aux libertés*

IV. MYCOLOGICAL INVESTIGATIONS (CONT'D)

CADRE RESERVE

BAL in case of pneumonia ☒ ***(store 5 ml of supernatant frozen)***

date of sampling : |__|__| day |__|__| month

84. Direct examination for capsulated yeasts: ☐ positive ☐ negative ☐ ND

85. CULTURE of *C. neoformans* : ☐ positive ☐ negative ☐ ND

86. Other pathogens isolated in the same sample, please detail

☐ bacteria ………… ☐ virus ……………

☐ fungus ……… ☐ parasite …………

OTHER BODY LOCALISATIONS

87a. Skin culture **:** ☐ positive ☐ negative ☐ ND

histology : ☐ positive ☐ negative ☐ ND

Date: |__|__| |__|__|

87b. **Lymph node**, culture **:** ☐ positive ☐ negative ☐ ND

histologie : ☐ positive ☐ negative ☐ ND

site : ……………………

88. Other (Please detail all body sites samples and cultures results):

……………………………………………………………………………………

……………………………………………………………………………………

……………………………………………………………………………………

……………………………………………………………………………………

V. INITIAL TREATMENT OF CRYPTOCOCCOSIS (D0)

CODE

DATE of initial prescription (**D0**) : |__|__| day |__|__| month ☐☐☐☐

89. Weight : ………………………… ☐☐☐

90. Height : ……………………………… ☐☐☐

91. Prescription of antifungal drugs : ☐ Yes ☐ No ☐

92. Amphotericin B deoxycholate ☐ yes mg/kg/d………….. ☐ no ☐ ☐,☐

93. Other formulation of amphotericin B (precise name and dose in mg/kg/j) : ☐

…………………………………………………………………………………

94. Flucytosine ☐ yes mg/kg/d …………..☐ No ☐ ☐☐☐

95. Fluconazole ☐ yes mg/d …………….. ☐ No ☐ ☐☐☐

96. Itraconazole ☐ Yes mg/d …………….. ☐ No ☐ ☐☐☐

97. Other (precise) : …………………………………………………. ☐

98. In case of intracranial hyperpressure:

☐ Repeated lumbar punctures ☐ shunt ☐ Nothing ☐

☐ steroids ☐ other …………..

99. Interval between first day of hospitalisation and onset of treatment (**in days**) : |__|__| ☐☐

Specific comments

………………………………………………………………………………………………

………………………………………………………………………………………………

………………………………………………………………………………………………

PLEASE CHECK THAT CULTURES HAVE BEEN DONE AND SAMPLES STORED FOR SUBSEQUENT STUDY

** articles 26, 27, 34 et 40 de la loi n°78-17 du 6/01/1978 relative à l'informatique, aux fichiers et aux libertés*

SIGNATURE :
DATE :

PLEASE SENT THE XEROX COPIE OF PAGES 2 TO 12 TO THE NCRM

Dr. F. Dromer - Dr. O. Lortholary
Crypto A/D study
National Reference Center for Mycoses
Institut Pasteur - 25, rue du Dr. Roux
75724 Paris cedex 15

** articles 26, 27, 34 et 40 de la loi n°78-17 du 6/01/1978 relative à l'informatique, aux fichiers et aux libertés*

FOLLOW-UP AT 2 WEEKS : Wk2 of the antifungal treatment

***DON'T FORGET TO CONTROL ALL SAMPLES THAT WERE INITIALLY CULTURE-POSITIVE AT D0 AND TO STORE SAMPLES** (indicated ☒)*

I. CLINICAL CHECK UP

CODE

100. Date : |__|__| day |__|__| month |__|__| year ☐☐☐☐

101. Still hospitalized ☐ Yes ☐ No ☐

Note all modifications (appearance of a sign initially absent should be checked "increased"

102. Fever : ☐ increased ☐ diminished ☐ stable ☐ none ☐

103. Meningism: ☐ increased ☐ diminished ☐ stables ☐ absent ☐

104. Abn. mental status: ☐ increased ☐ diminished ☐ stables ☐ absent ☐

105. Cranial nerve defect: ☐ increased ☐ diminished ☐ stable ☐ absent ☐

106.Motor defect: ☐ increased ☐ diminished ☐ stable ☐ absent ☐ GC☐

107. Death : ☐ yes, on |__|__| day |__|__| month ☐ non ☐ ☐☐☐☐

108. Death related to cryptococcosis : ☐ Yes ☐ if No, cause …………………
…………………………………………………………………………………………

II. MYCOLOGICAL INVESTIGATIONS (*C. neoformans*)

date : |__|__| **day** |__|__| **month** ☐☐☐☐

109. **CSF** ☒ (in case of initial meningoencephalitis) ☐

India ink : ☐ positive ☐ negative ☐ND ☐

culture: ☐ positive ☐ negative ☐ND ☐ ______

110. BLOOD ☒

culture : ☐ positive ☐ negative ☐ ND ☐ ______

111. URINES ☒ , culture : ☐ positive ☐ negative ☐ ND ☐ ______

112. BAL, culture : ☐ positive ☐ negative ☐ ND ☐ ______

113. Other sites : ……………………………………………………………… ☐

FOLLOW UP AT 2 WEEKS (Cont'd)

III. TREATMENT ***(Please note all events that occurred between D0 and Wk2)***

CADRE RESERVE

114. Modification of antifungals : ☐ Yes ☐ No ☐

115. Reason: ☐ failure ☐ toxicity ☐ systematic switch ☐ ☐

……… ☐☐☐☐

116. Date of the change |__|__| day |__|__| month

Details on each antifungal drug ***(if no change has been made, please fill only the cumulative dose received between D0 & Wk2)***

ANTIFUNGALS

117. **Amphotericin B** ☐ stopped ☐ new dosage : …… mg/kg/d ☐

Cumulative dose (**D0-wk2**): ………….. mg/d x ……….. days =………..mg ☐☐

Details if necessary ……………………………………………………………… ☐

118. Flucytosine ☐ stopped ☐ new dosage : …… mg/kg/d ☐

Cumulative dose (**D0-wk2**): ………..g/d x … days = ……….. g ☐☐☐

Details if necessary ……………………………………………………………… ☐

119. Fluconazole ☐ stopped ☐ new dosage : …… mg/d ☐

Cumulative dose (**D0-wk2**): ………..mg/d x ……….. days = …………..mg ☐☐☐

Details if necessary ……………………………………………………………… ☐

120. Itraconazole ☐ oui ☐ non new dosage: ………….. mg/j ☐

Cumulative dose (**D0-wk2**): ………..mg/j x ……….. days = …………..mg ☐☐☐

Details if necessary ……………………………………………………………… ☐

DON'T FORGET TO
***SIGN AND DATE THESE TWO PAGES* : ………………………………**

***SEND THEM TO THE NRCM* BY FAX (01 45 68 84 20)**

IF SAMPLES ARE STORED AT -20°C INSTEAD OF -80°C, PLEASE CONTACT US IMMEDIATELY at 33 1 40 61 36 90

** articles 26, 27, 34 et 40 de la loi n°78-17 du 6/01/1978 relative à l'informatique, aux fichiers et aux libertés*

FOLLOW-UP AT MONTH 3 (12 WEEKS)

***DON'T FORGET TO CONTROL ALL SAMPLES THAT WERE INITIALLY CULTURE-POSITIVE AT D0 AND TO STORE SAMPLES** (indicated ☒)*

I. CLINICAL CHECK UP

CODE

121. Date : |__|__| day |__|__| month |__|__| year ☐☐☐☐

122. Still hospitalized ☐ Yes ☐ No ☐

Note all modifications (appearance of a sign initially absent should be checked "increased")

123. Fever : ☐ increased ☐ diminished ☐ stable ☐ none ☐

124. Meningism : ☐ increased ☐ diminished ☐ stable ☐ absent ☐

125. Abn. mental status ☐ increased ☐ diminished ☐ stable ☐ absent ☐

126. Cranial nerve defect: ☐ increased ☐ diminished ☐ stable ☐ absent ☐

127. Motor defect: ☐ increased ☐ diminished ☐ stable ☐ absent ☐

128. Clinical cure (disparition of all abnormal signs) : ☐ Yes ☐ No ☐

129. Neurological sequellae ☐ yes (details)............................. ☐ No … ☐

129. Deaths : ☐ Yes on |__|__| day |__|__| month ☐ No ☐☐☐☐

130. Death related to cryptococcosis : ☐ Yes ☐ No, cause ....
.... ☐ |____|

II. MYCOLOGICAL INVESTIGATIONS (*C. neoformans*)

date : |__|__| **day** |__|__| **month** ☐☐☐☐

131. **CSF** ☒ (if initial meningoencephalitis) ☐

India ink : ☐ positive ☐ negative ☐ ND ☐

Culture: ☐ positive ☐ negative ☐ ND ☐ ______

132. BLOOD ☒ culture : ☐ positive ☐ negative ☐ ND ☐ ______

133. URINES ☒ culture : ☐ positive ☐ negative ☐ ND ☐ ______

134. BAL, culture : ☐ positive ☐ négative ☐ ND ☐ ______

135. Other body sites : .... ☐ ______

** articles 26, 27, 34 et 40 de la loi n°78-17 du 6/01/1978 relative à l'informatique, aux fichiers et aux libertés*

FOLLOW-UP AT 3 MONTHS (Cont'd)

III. TREATMENT ***(note all events that occurred during Wk2 and Mo3)***

CADRE

136. Modification of antifungals prescribed: ☐ Yes ☐ No ☐

137. Reasons: ☐ Failure ☐ toxicity ☐ systematic switch ☐ ☐

……… ☐☐☐☐

138. Date of modification |__|__| day |__|__| month

Details on each antifungal drug ***(if no change has been made, please fill only the cumulative dose received between Wk2 & Mo3)***

CHANGES FOR

139. Amphotericin B ☐ stopped ☐ new dosage : …… mg/kg/d ☐

Cumulative dose (**Wk2 – Mo3**): ……………mg/d x ……….. days =………..mg ☐☐

Details if necessary ………………………………………………………………… ☐

140. Flucytosine ☐ stopped ☐ new dosage: …… mg/kg/d ☐

Cumulative dose (**Wk2 – Mo3**): ………..g/d x … days = ……….. g ☐☐☐

Details if necessary ………………………………………………………………… ☐

141. Fluconazole ☐ stopped ☐ new dosage : …… mg/d ☐

Cumulative dose (**Wk2 – Mo3**): ………..mg/d x ……….. days = ……………mg ☐☐☐

Details if necessary ………………………………………………………………… ☐

142. Itraconazole ☐ oui ☐ non dosage : …………… mg/d ☐

Cumulative dose (**Wk2 – Mo3**): ………..mg/d x ……….. days = ……………mg ☐☐☐

Details if necessary ………………………………………………………………… ☐

DON'T FORGET TO
SIGN AND DATE THESE TWO PAGES : ………………………………

SENT THEM TOGETHER WITH A COPY OF THE ENTIRE QUESTIONNAIRE BY MAIL TO THE NRCM

** articles 26, 27, 34 et 40 de la loi n°78-17 du 6/01/1978 relative à l'informatique, aux fichiers et aux libertés*
