# Supplementary material for: Determinants of Disease Presentation and Outcome during Cryptococcosis: The CryptoA/D Study
Source: PLoS Med. 2007 Feb 6;4(2):e21. doi: 10.1371/journal.pmed.0040021 (PMC1808080; doi:10.1371/journal.pmed.0040021)
Supplement: Alternative Language Abstract S1 — (39 KB DOC) [file pmed.0040021.sd002.doc]

**RESUME**

**Contexte :** la cryptococcose est une infection opportuniste menaçant le pronostic vital chez des patients infectés ou non par le VIH. Les données disponibles sur la présentation clinique et les recommandations thérapeutiques actuelles dérivent essentiellement d'essais thérapeutiques menés avant l'introduction des antirétroviraux hautement actifs chez des patients ayant une méningo-encéphalite cryptococcique, et prennent peu en compte les infections extra-méningées ou les infections par le sérotype D de *Cryptococcus neoformans* par opposition au sérotype A.

**Méthodes et résultats :** une étude prospective multicentrique, CryptoA/D a été mise en place en France (1997-2001) pour analyser les facteurs influençant la présentation clinique et l'évolution de l'infection sans biais d'inclusion dans un essai thérapeutique. Des 230 patients inclus, 177 (77%) étaient infectés par le VIH, 50 (22%) étaient des femmes, et 161 (72,5%) étaient infectés par le sérotype A. Sur la base des résultats des cultures faites au moment du diagnostic, l'infection était plus sévère chez les hommes, les patients infectés par le VIH et ceux infectés par le sérotype A. Les paramètres indépendamment associés à l'échec mycologique au bout de 2 semaines de traitement antifongiques, et ce quelque soit le statut VIH, étaient l'existence d'une dissémination lors du bilan initial (OR=2.4 [1.2-4.9]), un titre antigénique élevé (>1:512) dans le sérum (OR=2.6 [1.3-5.4]), et l'absence de 5-fluorocytosine en traitement d'induction (OR=3.8 [1.9-7.8]). La survie à trois mois était significativement raccourcie chez les patients ayant initialement un bilan clinique neurologique anormal, une imagerie cérébrale anormale, ou une hémopathie.

**Conclusion:** Le statut VIH, le sexe du patient et le sérotype infectant sont des paramètres majeurs influençant la présentation clinique et l'évolution de la cryptococcose. Nous proposons une modification des recommandations thérapeutiques actuelles pour la prise en charge initiale des patients atteints de cryptococcose sur la base de l'évaluation systématique de la charge fongique initiale.
